# Compound hot–dry events greatly prolong the recovery time of dryland ecosystems

**DOI:** 10.1093/nsr/nwae274

**Published:** 2024-08-09

**Authors:** Ying Yao, Bojie Fu, Yanxu Liu, Yao Zhang, Jingyi Ding, Yan Li, Sha Zhou, Jiaxi Song, Shuai Wang, Changjia Li, Wenwu Zhao

**Affiliations:** State Key Laboratory of Earth Surface Processes and Resource Ecology, Faculty of Geographical Science, Beijing Normal University, Beijing 100875, China; State Key Laboratory of Urban and Regional Ecology, Research Center for Eco-Environmental Sciences, Chinese Academy of Sciences, Beijing 100085, China; State Key Laboratory of Earth Surface Processes and Resource Ecology, Faculty of Geographical Science, Beijing Normal University, Beijing 100875, China; Sino-French Institute for Earth System Science, College of Urban and Environmental Sciences, Peking University, Beijing 100871, China; State Key Laboratory of Earth Surface Processes and Resource Ecology, Faculty of Geographical Science, Beijing Normal University, Beijing 100875, China; State Key Laboratory of Earth Surface Processes and Resource Ecology, Faculty of Geographical Science, Beijing Normal University, Beijing 100875, China; State Key Laboratory of Earth Surface Processes and Resource Ecology, Faculty of Geographical Science, Beijing Normal University, Beijing 100875, China; State Key Laboratory of Earth Surface Processes and Resource Ecology, Faculty of Geographical Science, Beijing Normal University, Beijing 100875, China; State Key Laboratory of Earth Surface Processes and Resource Ecology, Faculty of Geographical Science, Beijing Normal University, Beijing 100875, China; State Key Laboratory of Earth Surface Processes and Resource Ecology, Faculty of Geographical Science, Beijing Normal University, Beijing 100875, China; State Key Laboratory of Earth Surface Processes and Resource Ecology, Faculty of Geographical Science, Beijing Normal University, Beijing 100875, China

**Keywords:** recovery time, drought severity, drought duration, vegetation response, vapor pressure deficit, high temperature

## Abstract

Compound hot–dry events cause more severe impacts on terrestrial ecosystems than dry events, while the differences in recovery time (ΔRT) between hot–dry and dry events and their contributing factors remain unclear. Both remote sensing observations and eddy covariance measurements reveal that hot–dry events prolong the recovery time compared with dry events, with greater prolongation of recovery time in drylands than in humid regions. Random forest regression modeling demonstrates that the difference in vapor pressure deficit between hot–dry and dry events, with an importance score of 35%, is the major factor contributing to ΔRT. The severity of stomatal restriction exceeds that of non-stomatal limitation, which restricts the vegetation productivity that is necessary for the recovery process. These results emphasize the negative effect of vapor pressure deficit on vegetation recovery during hot–dry events and project an extension of drought recovery time considering elevated vapor pressure deficit in a warming world.

## Teaser

A large increase in VPD during compound hot–dry events dominated the greater prolongation of recovery time in dryland ecosystems.

## INTRODUCTION

Under global warming, the probability and severity of compound hot–dry events have increased substantially [1–3]. It is widely reported that hot–dry events cause more serious losses than droughts without heat (precipitation-deficit droughts) [4,5]. For example, in Spain, moderate compound dry and hot conditions increased the likelihood of productivity loss by 8%–11% compared with that under moderate dry conditions [6]. In addition to the negative impacts on productivity, hot–dry events may also increase the mortality of vegetation and induce detrimental and sudden changes in the structure or function of ecosystems [7], which reduce the structural and functional stability of ecosystems. Given the importance of recovery capacity in maintaining ecosystem stability [8], it is critical to explore the recovery of ecosystems after hot–dry events.

Recovery time—the length of time it takes for ecosystems to return to normal states—is one of the important indicators used to measure the recovery of ecosystems from droughts [9,10]. Previous research has reported that the recovery time for most ecosystems is between 2 and 8 months [11,12], but these drought recovery time assessments did not distinguish between hot–dry events and dry events. Considering that hot–dry events usually caused more severe vegetation losses than dry events, ecosystems are expected to require a longer time to recover from hot–dry events. However, a quantitative assessment of the recovery time of ecosystems from hot–dry events is lacking. With the increasing frequency of hot–dry events [13], ecosystems with long recovery times may suffer another hot–dry event without full recovery, resulting in superimposed damage to ecosystems. Therefore, it is crucial to reveal the recovery dynamics of ecosystems from hot–dry events, contributing to the management and protection of ecosystems.

Post-drought recovery largely depends on the vegetation productivity for root growth, rebuilding of lost leaf area and regeneration of non-structural carbohydrate [14]. These processes are inseparable from efficient carbon and water cycles [15]. When vegetation is in water deficit (high vapor pressure deficit and low soil moisture), it regulates stomatal conductance to maximize carbon gains while reducing water loss [16]. Previous studies have reported that soil moisture effects dominated dryness stress on ecosystems and determined the recovery time of ecosystems from droughts [11,16]. However, when it comes to the recovery time of hot–dry events, there is no evidence proving the relative importance of low soil moisture and high vapor pressure deficit, which inhibits our understanding of ecosystem recovery from these two types of droughts. Given the significant increase in vapor pressure deficit under hot–dry events, is the relative importance of vapor pressure deficit for recovery time greater under such conditions? In addition, under hot–dry events, high temperature could reduce ribulose-1,5-bisphosphate (RuBP) content and Rubisco activity, and such non-stomatal limitations inhibit various physiological activities and ultimately impact vegetation recovery [17]. However, the performances of stomatal and non-stomatal limitations under these two types of droughts are unclear.

To address the abovementioned research gaps, this study explored the difference in the recovery time between hot–dry and dry events, and analysed the factors contributing to the difference in recovery time. We conducted this research in the following three parts. First, a hot–dry event refers to a dry event accompanied by a hot event and a dry event is not accompanied by hot event (see ‘Methods’). We determined the difference in recovery time between hot–dry and dry events based on the normalized difference vegetation index (NDVI), leaf area index (LAI), gross primary productivity (GPP) and vegetation optical depth (VOD). Second, we applied a random forest regression model to quantify the relative importance of contributing factors to the difference in recovery time. Third, we comparatively analysed the stomatal activities (canopy conductance, *G*_c_) and non-stomatal activities (maximum photosynthetic assimilation rate, *A*_max_) under hot–dry and hot conditions based on eddy covariance measurements to understand the recovery process from vegetation physiology. Our results indicate that the difference in the recovery time between hot–dry and dry events was more protracted in drylands than in humid regions, and the main factor was the difference in vapor pressure deficit. This study highlights that the negative effects of a high vapor pressure deficit on ecosystem recovery outweigh those of low soil moisture under hot–dry events. With global warming, the vapor pressure deficit is expected to increase [18], causing damage to a wider range of ecosystems, especially in drylands.

## RESULTS AND DISCUSSION

### Difference in recovery time of ecosystems between hot–dry and dry events

**Figure 1.**
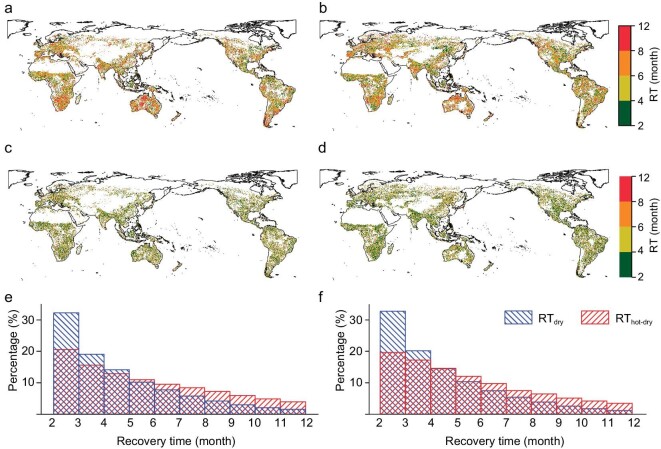
Recovery time of ecosystems from hot–dry and dry events. (a, b) Recovery time of ecosystems from hot–dry events (RT_hot–dry_) based on the NDVI and LAI, respectively. (c, d) Recovery time of ecosystems to dry events (RT_dry_) based on the NDVI and LAI, respectively. (e, f) Percentage of different recovery times based on the NDVI and LAI, respectively. Regions with sparse vegetation or no droughts are masked with white. Review drawing number: GS京(2024)1579.

Compared with dry events, hot–dry events extend the recovery time by >1 month (Fig. 1). The global average recovery time of ecosystems from hot–dry and dry events was 5.43 and 4.23 months, respectively, based on the NDVI (Fig. 1a and c), consistently with the result obtained from LAI with average recovery time of 5.18 and 4.1 months, respectively (Fig. 1b and d). More than 80% of ecosystems recover from dry events within 6 months and ∼10% of ecosystems take >8 months to recover (Fig. 1c–f). Ecosystem recovery from hot–dry events tends to be more difficult—specifically, 20% of ecosystems need ≥8 months to recover (Fig. 1a, b, e and f), including those in southern South America, the Mediterranean region, southern Africa and Australia (Fig. 1a and b). This result suggests that declined moisture content (from humid region to dryland) reduces ecosystem resilience (Fig. S1) [9,19]. In addition to moisture availability, biological factors, such as canopy height, can also affect recovery after drought. Using global forest canopy height data [20], we discussed the correlation coefficients between recovery time and canopy height at the dryland, humid region and global scales, respectively. There are significant positive correlation coefficients between recovery time and canopy height (Fig. S2b). These results suggest that the recovery time increases with canopy height, which is supported by previous studies of drought-related tree mortality which have shown that large trees find it more difficult to recover and have higher mortality rates than small trees [21,22]. This could be explained by the fact that larger trees tend to take longer to produce sufficient material to repair the productivity loss (growth decline) caused by droughts [21].

**Figure 2.**
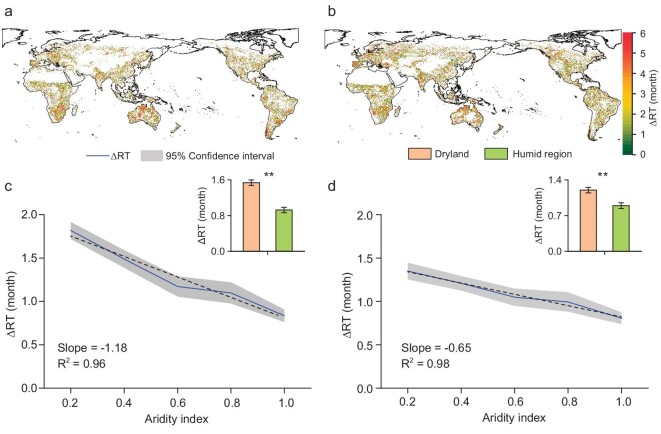
Difference in recovery time between hot–dry and dry events. (a, b) Spatial patterns of the difference in recovery time between hot–dry and dry events (ΔRT = RT_hot–dry_ – RT_dry_) based on the NDVI and LAI, respectively. (c, d) ΔRT under different aridity levels (aridity index, the ratio of precipitation to potential evapotranspiration) based on the NDVI and LAI, respectively. Asterisks indicate statistically significant differences (**P < 0.05). Review drawing number: GS京(2024)1579.

We use the difference in recovery time between hot–dry and dry events (ΔRT) to characterize the prolongation of recovery time caused by hot–dry events compared with dry events, in which a larger ΔRT means a greater prolongation of recovery time during hot–dry events. Along different aridity gradients, ΔRT is always >0 (Fig. 2c and d), meaning that it is more difficult for ecosystems to recover from hot–dry events than from dry events. There is no doubt that there is longer time for ecosystems to recover from hot–dry than from dry events, given that the hot–dry events have greater drought severity and more severe moisture deficits (Fig. S3). Notably, ΔRT shortens with an increase in the aridity index, and the slope of the linear regression of ΔRT and the aridity index is –1.18 and –0.65 based on the NDVI and LAI, respectively (Fig. 2c and d), indicating that the prolongation of recovery time caused by hot–dry events is more severe in drier regions. In humid regions, ΔRT based on the NDVI and LAI is 0.93 and 0.90 months, respectively; however, ΔRT reaches 1.54 and 1.20 months in drylands (Fig. 2c and d, and Fig. S1) and even exceeds 4 months in Australia, Southern Africa, Southern South America and the Mediterranean region (Fig. 2a and b).

Consistently with NDVI and LAI, microwave VOD data reveal that hot–dry events lead to longer recovery time than dry events (Fig. S4a–c) and greater prolongation of recovery time in drylands than in humid regions (Fig. S4d). This suggests that hot–dry events cause more serious damage to biomass and carbon stock in dryland than in humid regions [23]. Notably, recovery time calculated using VOD (carbon stock) data is the longest among these three data sets (Fig. 1 and Fig. S4). Specifically, for hot–dry events, the recovery times based on NDVI, LAI and VOD are 5.43, 5.18 and 5.66 months, respectively (Fig. 1a and b, and Fig. S4c). For dry events, the recovery times based on NDVI, LAI and VOD are 4.23, 4.1 and 4.26 months, respectively (Fig. 1c and d, and Fig. S4c). This result implies that biomass and carbon stock recover more slowly after disturbance than leaf-scale area or greenness [24,25].

Considering that human activities may affect recovery time, we supplemented the recovery time of ecosystems from hot–dry and dry events under different human footprints. Under a low human footprint (human footprint is below the global average), the recovery time of ecosystems from hot–dry and dry events is 5.20 and 4.04 months (Fig. S5a), respectively, and ΔRT in dryland and humid regions is 1.22 and 1.07 months (Fig. S5b), respectively. Under a high human footprint (human footprint is above the global average), the recovery time of ecosystems from hot–dry and dry events is 5.15 and 4.18 months (Fig. S5c), respectively, and ΔRT in dryland and humid regions is 1.14 and 0.84 months (Fig. S5d), respectively. This result indicates that the finding of greater prolongation of recovery time under hot–dry events in drylands than in humid regions is robust under different human footprints.

### Contributing factors of ΔRT

**Figure 3.**
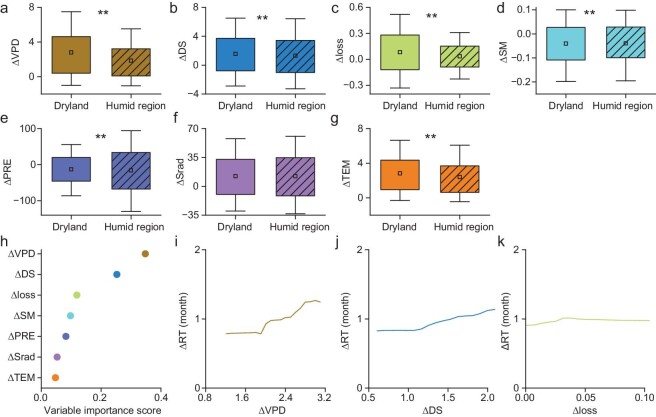
Contributing factors to the difference in recovery time between hot–dry and dry events. (a–g) ΔVPD, ΔDS, Δloss, ΔSM, ΔPRE, ΔSrad and ΔTEM. (h) Variable importance score based on the random forest regression model. (i–k) Partial dependence plots of ΔRT with the three most important variables (ΔVPD, ΔDS and Δloss). ΔVPD, ΔSM, ΔPRE, ΔSrad and ΔTEM represent the difference in vapor pressure deficit, soil moisture, precipitation, shortwave radiation and temperature during the recovery period between hot–dry and dry events. ΔDS represents drought severity indicated by the sum of SPI during drought duration. Δloss represents the difference in vegetation loss. Asterisks indicate statistically significant differences (**P < 0.05).

We attribute the ΔRT between drylands and humid regions to the differences in climatic factors during the recovery period, drought severity and vegetation loss (see ‘Methods’). The average ΔVPD (difference in vapor pressure deficit) anomaly between hot–dry and dry events is 2.8 and 1.85 in drylands and humid regions, respectively (Fig. 3a). This could be explained by the high temperature in the compound hot–dry environment increasing the atmospheric moisture demand and exacerbating soil evaporation and vegetation transpiration [26,27]. A previous study revealed that the evaporation rate could increase by 0.07 mm/day for each 1°C increase in soil temperature [28], leading to a decline in soil moisture [28,29]. When the soil water drops to relatively low levels, evaporation and transpiration are water-limited [30], resulting in less moisture transported to the atmosphere, especially in drylands [31]. Thus, there are substantial increases in vapor pressure deficit in drylands [32].

The average ΔDS (difference in drought severity) is 1.55 and 1.31 in drylands and humid regions, respectively, indicating that hot–dry events cause greater drought severity than dry events, and drylands are more severely impacted than humid regions (Fig. 3b). Vegetation losses caused by hot–dry events are greater than those caused by dry events, and the average Δloss is 0.08 and 0.04 in drylands and humid regions, respectively (Fig. 3c). Random forest regression modeling showed that ΔVPD is the most important contributing factor to ΔRT, followed by ΔDS and Δloss (Fig. 3h). The partial dependence plot reveals that ΔRT increases with increasing ΔVPD, ΔDS and Δloss (Fig. 3i–k) and these results suggest that the greater ΔVPD, ΔDS and Δloss in drylands than in humid regions contribute to the variation in ΔRT along the aridity gradient.

Although the contribution of ΔSM (difference in soil moisture) to ΔRT is lower than that of ΔVPD, ΔDS and Δloss (Fig. 3h), the significant negative correlation and partial correlation coefficients between ΔRT and ΔSM show that a lower ΔSM prolongs recovery time (Fig. 3d and Fig. S6). The ΔTEM (difference in temperature) in drylands is significantly higher than that in humid regions (Fig. 3g), but its contribution to ΔRT is lower than that of ΔVPD and ΔSM (Fig. 3h and Fig. S6). This could be explained by the fact that soil moisture and vapor pressure deficit have a direct impact on the vegetation recovery process by affecting the carbon–water cycle (vegetation roots absorb water from soil, and stomata regulate carbon uptake and water loss) [15], while temperature indirectly affects the recovery time by affecting soil moisture evaporation, vegetation transpiration, etc. [33].

### Stomatal and non-stomatal limitations caused by hot–dry and dry events

**Figure 4.**
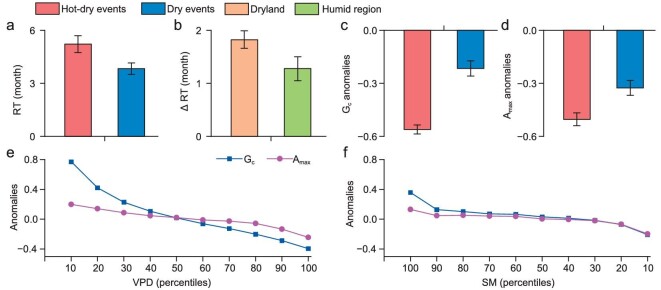
Response of vegetation to hot–dry and dry events based on eddy covariance measurements from the FLUXNET2015 dataset. (a) Comparison between recovery time of ecosystems from hot–dry and dry events. (b) Difference in recovery time between ecosystems from hot–dry and dry events. (c) *G*_c_ anomalies under hot–dry and dry events. (d) *A*_max_ anomalies under hot–dry and dry events. (e) Response curves of *G*_c_ anomalies and *A*_max_ anomalies related to increased vapor pressure deficit. (f) Response curves of *G*_c_ anomalies and *A*_max_ anomalies related to decreased soil moisture.

Post-drought recovery is largely dependent on the vegetation productivity for reconstructing vegetation loss caused by drought [14] and it is crucial to eliminate the stomatal and non-stomatal limitations for the efficient progress of photosynthesis. We evaluate the recovery time of ecosystems from hot–dry and dry events based on GPP, temperature and precipitation from the FLUXNET2015 data set, and analyse the stomatal and non-stomatal anomalies under hot–dry and dry events based on canopy conductance (*G*_c_) and the maximum photosynthetic assimilation rate (*A*_max_) (see ‘Methods’). Consistently with remote sensing, eddy covariance measurements show that hot–dry events cause longer recovery time than dry events (Fig. 4a) and greater prolongation of recovery time in drylands than in humid regions (Fig. 4b). Compared with dry events, hot–dry events cause more severe stomatal and non-stomatal limitations (Fig. 4c and d). This result means greater damage to photosynthesis, explaining the longer recovery time from hot–dry than from dry events.

Notably, the *G*_c_ anomalies of hot–dry events are 2.55 times those of dry events, and the *A*_max_ anomalies of hot–dry events are 1.52 times those of dry events, meaning that hot–dry events lead to more severe stomatal restriction than non-stomatal restriction (Fig. 4c and d). This result is supported by an analysis of stomatal and non-stomatal limitations in mature deciduous tree species. It was found that, when considered independently from leaf age, the response of trees to drought was dominated by stomatal limitations, accounting for ∼75% of the total limitation [34]. Under moisture pressure, plants close their stomata to avoid the collapse caused by hydraulic failure due to xylem embolism [35]. Previous studies have reported that vapor pressure deficit directly affects vegetation transpiration through water potential [35,36] and the response of stomatal closure to the water potential of leaves or the canopy is timely and sensitive [37–40]. Stomatal limitations are more sensitive to both vapor pressure deficit and soil moisture than are non-stomatal limitations (Fig. 4e and f). Therefore, there are more severe stomatal limitations due to lower soil moisture and higher vapor pressure deficit under hot–dry events (Fig. S3).

## CONCLUSION AND IMPLICATIONS

Considering the increasing hot–dry events under global warming (Figs S7 and S8), a wider range of ecosystems may suffer from repeated hot–dry events when they have not fully recovered. Clarifying the process of ecosystem recovery from hot–dry events is conducive to ecosystem management and conservation. Here, our study presents comprehensive evidence of the prolonged recovery time of vegetation in response to hot–dry events compared with dry events and the driving mechanisms based on global evidence from both remote sensing observations and eddy covariance measurements. We found that the recovery time from hot–dry events exceeds 5 months, which is more than that from dry events (∼4 months), and the difference in recovery time between hot–dry and dry events is exacerbated with intensified dryness. Spatially, ΔRT is >1.5 months in drylands, which is significantly longer than that in humid regions. The intensification of vapor pressure deficit in drylands under hot–dry events is the main factor contributing to the greater prolongation of recovery time in drylands.

The longer recovery time in drylands is indicative of high fragility during compound hot–dry events and the delayed recovery may bring about risks of degradation due to the inability to recover from repeated droughts [9,41]. It should be noted that glacier meltwater is one of the sources of water in arid regions [42] and precipitation alone could not adequately reflect local water deficits. This may lead to uncertainty in the recovery time assessment in the arid areas. High-temperature environments could exacerbate the glacial meltwater to compensate for the lack of precipitation [43]. More glacial meltwater and soil water monitoring in arid areas would facilitate regional drought assessment.

The intensification of vapor pressure deficit in drylands under hot–dry events dominates greater prolongation of recovery time in drylands than in humid regions. This finding updates the previous notion that soil moisture determines dryness stress and dominates drought recovery time [11,16], providing new insights for the vegetation recovery process of dryland ecosystems during compound hot–dry events. The significant increase in vapor pressure deficit in drylands due to high temperatures leads to severe stomatal limitations [36,38], which restrict the vegetation productivity necessary for the recovery process, resulting in longer recovery times. Considering global warming and decreasing terrestrial relative humidity [44], terrestrial ecosystems are expected to face increasing drought risk and recovery pressure, which may pose risks to the carbon sinks of ecosystems and challenge climate mitigation based on natural climate solutions.

## METHODS

### Drought index and vegetation index

We used the standardized precipitation index (SPI), which is a universal drought index, to identify dry conditions [45]. Notably, we did not use the standardized precipitation evapotranspiration index calculated based on monthly precipitation and evapotranspiration impacted by temperature because we needed to define compound hot–dry events and droughts without hot events separately [46]. SPI at 3 months—a short- and medium-term drought indicator—was selected to explore the impact of drought on vegetation in this study [47,48]. We selected the NDVI and LAI from the Global Inventory Monitoring and Modeling System 3g as proxies for characterizing the dynamics of vegetation growth [47,49,50].

### Recovery time of ecosystems in response to hot–dry events and dry events

To accurately quantify ecosystem recovery time [51,52], the vegetation data used needed to be devoid of seasonal cycles and long-term trends (Fig. S9). We defined hot–dry events as the co-occurrence of the following three criteria (Fig. S10): (i) SPI was below –1 and lasted for ≥2 months [52,53]; (ii) a hot event was defined as an average temperature for two consecutive months exceeding the 90th percentile of temperature over the same period [2]; (iii) detrended vegetation data were below –0.1 SD (Fig. S10). Meanwhile, we determined the dry events according to three criteria: (i) SPI was below –1 and lasted for ≥2 months; (ii) there were no two consecutive months with temperatures above the 90th percentile of the same period; (iii) detrended vegetation data were below –0.1 SD. Recovery time was defined as the time it took for vegetation to return to its normal state from the maximum loss (Fig. S10). According to the aridity index (the ratio of precipitation to potential evapotranspiration) [54], we further determined the differences in recovery time (ΔRT) between hot–dry and dry events under different aridity levels. We supplemented the assessment of recovery time based on a negative vegetation anomaly threshold of –0.5 SD (Fig. S11).

### Contributing factors of ΔRT between hot–dry and dry events

We explained the ΔRT between hot–dry and dry events according to the following three aspects: DS, vegetation loss and climatic factors during the recovery period caused by drought [12,55,56]. More details are available in the Supplementary material.

### Stomatal and non-stomatal limitations caused by hot–dry and dry events derived from eddy covariance measurements

Productivity loss or greenness decline of vegetation results from stomatal and non-stomatal (maximum photosynthetic rate) limitations caused by droughts [38]. Stomatal limitation refers to the decline in photosynthesis caused by the partial closure of stomata during drought to save water [57] and non-stomatal restriction mainly refers to the decline in photosynthesis caused by non-stomatal factors such as the degradation of chloroplasts [58] and the declines in RuBP content and Rubisco activity [17]. We characterized the stomatal and non-stomatal limitation under hot–dry and dry events by using canopy conductance (*G*_c_) and the maximum photosynthetic assimilation rate (*A*_max_) derived from eddy covariance measurements from the FLUXNET2015 Tier 1 data set (Figs S12 and S13) [34,38,59–63].

## DATA AND CODE AVAILABILITY

The LAI data set is available at http://www.mdpi.com/2072-4292/5/2/927. NDVI 3gv1 is available at http://poles.tpdc.ac.cn/en/data/9775f2b4-7370-4e5e-a537-3482c9a83d88/. The FLUXNET2015 data set is available at https://fluxnet.org/data/fluxnet2015-dataset/. The microwave-based VOD data are available at http://files.ntsg.umt.edu/data/LPDR_v2/. Precipitation, potential evapotranspiration, actual vapor pressure and temperature data are from the Climatic Research Unit gridded Time Series (CRU TS 4.05), available at https://crudata.uea.ac.uk/cru/data/hrg/. Root soil moisture data are available at https://www.gleam.eu/. Shortwave radiation data are available at https://cds.climate.copernicus.eu/cdsapp#!/dataset/reanalysis-era5-land-monthly-means?tab=overview. Human footprint data are available at https://figshare.com/articles/figure/An_annual_global_terrestrial_Human_Footprint_dataset_from_2000_to_2018/16571064. Global forest canopy height data are available at https://glad.umd.edu/dataset/gedi/.

Data preprocessing and the calculation of recovery time and *G*_c_ were performed in MATLAB (R2020b), *A*_max_ was calculated in R.4.0.2, random forest regression was performed in Python 3.9.0 and the figures were produced in Origin 2023.

## Supplementary Material

nwae274_Supplemental_File
