# Supplementary material for: Compound hot–dry events greatly prolong the recovery time of dryland ecosystems
Source: Natl Sci Rev. 2024 Aug 9;11(10):nwae274. doi: 10.1093/nsr/nwae274 (PMC11409867; doi:10.1093/nsr/nwae274)
Supplement: nwae274_Supplemental_File [file nwae274_supplemental_file.docx]

Compound hot-dry events greatly prolong the recovery time of dryland ecosystems

Ying Yao^1^, Bojie Fu^1*^, Yanxu Liu^1^, Yao Zhang^2^, Jingyi Ding^1^, Yan Li^1^, Sha Zhou^1^, Jiaxi Song^1^, Shuai Wang^1^, Changjia Li^1^, Wenwu Zhao^1^

1. State Key Laboratory of Earth Surface Processes and Resource Ecology, Faculty of Geographical Science, Beijing Normal University, Beijing, China

2. Sino-French Institute for Earth System Science, College of Urban and Environmental Sciences, Peking University, Beijing, China

*Corresponding author. Email: [bfu@rcees.ac.cn](mailto:bfu@rcees.ac.cn)

# Methods

## Drought index and vegetation index

We used the standardized precipitation index (SPI), a universal drought index, to identify dry conditions [1]. Notably, we did not use the standardized precipitation evapotranspiration index (SPEI) calculated based on monthly precipitation and evapotranspiration impacted by temperature [2] because we needed to define compound hot-dry events and droughts without hot events separately. SPI was calculated based on the probability of precipitation, representing the degree of standardized deviation. Positive and negative SPI values indicated wet and dry conditions, respectively, and a larger absolute value of the SPI indicated a larger deviation from the normal state. SPI can be calculated at multiple timescales, such as 1 month and 3, 6, 12, 24 and 48 months, to reflect short- or long-term drought characteristics. SPI at 3 months, a short- and medium-term drought indicator, was selected to explore the impact of drought on vegetation in this study [3]. We calculated the SPI from 1982 to 2016 using monthly precipitation with a spatial resolution of 0.5°×0.5° from CRU TS v.4.05 based on the gamma distribution in MATLAB (R2022b) [4].

We selected the NDVI and LAI from the Global Inventory Monitoring and Modeling System (GIMMS) 3g as proxies to characterize the dynamics of vegetation growth [3,5,6]. To match the temporal and spatial resolutions of the SPI, we obtained monthly NDVI and LAI data by the maximum value composite and then used the bilinear resampling technique to obtain data with a spatial resolution of 0.5°×0.5°. The time spans of the NDVI and LAI were 1982-2015 and 1982-2016, respectively.

## Recovery time of ecosystems in response to hot-dry events and dry events

To accurately quantify ecosystem recovery time, the vegetation data used should be devoid of seasonal cycles and long-term trends. First, we abandoned the pixels with monthly vegetation values below 0.1 to exclude bare ground and vegetation that was completely defoliated in winter and then obtained deseasonalised NDVI and LAI by removing the average value of the NDVI and LAI from their time series to avoid the impact of seasonal cycles [7,8]. Taking the NDVI as an example, for the deseasonalised time series, a value above (below) 0 indicated that the vegetation state in the current month was in a positive (negative) anomaly compared with the multiyear average state. We presented the raw value of the NDVI and deseasonalised NDVI values during 30 consecutive months, and the raw value of the NDVI in February during three consecutive years was 0.28, 0.19 and 0.26, respectively; the deseasonalised NDVI was 0.02, -0.06 and 0.007, respectively (Fig. S9). Such treatment can identify vegetation anomalies in nongrowing seasons and avoid the impact of partial defoliation in nongrowing seasons on the identification of recovery. The “detrend” function in MATLAB (R2020b) was used to subtract the linear trends of deseasonalised NDVI and LAI to focus on the fluctuations in vegetation suffering from droughts. A slight vegetation decline caused by drought tends to recover immediately [9], and such events (highlighted in purple in Fig. S10b) do not require a recovery time assessment. Therefore, we exclude events that the detrended NDVI fall between 0 and 0.1 standard deviation (SD) during droughts, and select the detrended NDVI of less than -0.1 SD for evaluating recovery time.

We defined hot-dry events as the co-occurrence of the following three criteria (Fig. S10). (1) SPI was below -1 and lasted at least two months [10]. (2) A hot event was defined as an average temperature for 2 consecutive months exceeding the 90th percentile of temperature over the same period [11]. (3) Detrended vegetation data were below -0.1 SD. Meanwhile, we determined the dry events according to three criteria. (1) SPI was below -1 and lasted at least two months. (2) There were no 2 consecutive months with temperatures above the 90th percentile of the same period. (3) Detrended vegetation data were below -0.1 SD. Taking hot-dry event as an example, we selected events that met the above three conditions to identify the recovery time, which was defined as the time it took for vegetation to return to its normal state from the maximum loss (Fig. S10). We calculated the recovery time of ecosystems in response to dry events according to the same method for comparatively analysing the difference in recovery time between hot-dry and dry events. According to the aridity index (AI, the ratio of precipitation to potential evapotranspiration) [12], we further determined the differences in recovery time (ΔRT) between hot-dry and dry events under different aridity levels and characterized the relationship between ΔRT and aridity index by using linear regression. To enhance robustness of recovery time assessments under different thresholds of negative anomaly in vegetation, we supplemented the assessment of recovery time based on a negative vegetation anomaly threshold of -0.5 SD and presented the difference in recovery time between hot-dry and dry events (Fig. S11).

## Contributing factors of ΔRT between hot-dry and dry events

Drought severity (DS), vegetation loss and climatic factors during the recovery period caused by drought were considered important factors contributing to recovery time [13,14]; therefore, we explained the ΔRT between hot-dry and dry events according to the following three aspects: one was the difference in DS between hot-dry and dry events. DS was calculated as the sum of SPI values during the drought duration (Fig. S10a). The second was the difference in vegetation loss between hot-dry and dry events. Vegetation loss was defined as the difference between the minimum detrended vegetation index and the normal state (Fig. S10b). Third was the differences in climatic factors during the recovery period between hot-dry and dry events. Climatic variables included soil moisture from the Global Land Evaporation Amsterdam Model (GLEAM v3) dataset, vapor pressure deficit, temperature from CRU TS v.4.05, precipitation from CRU TS v.4.05, and shortwave radiation from ERA5. Vapor pressure deficit was calculated based on the actual vapour pressure (AVP) from CRU TS v.4.05 and temperature according to Equations 1-3:

$SVP =6.112*\left( 1+7*{10}^{-4}+3.46*{10}^{-6}*P_{mst} \right)*e^{\frac{17.67*T_{a}}{T_{a}+243.5}}$ (1)

$P_{mst}=1013.25*{(\frac{273.16+T_{a}}{273.16+T_{a}+0.0065*Z})}^{5.625}$ (2)

$VPD=SVP-AVP$ (3)

where *SVP* is the saturated vapour pressure (kPa), *T_a_* is the land air temperature (℃), *z* is the altitude (m), and *VPD* is the vapor pressure deficit.

According to the processing method of vegetation indices, we first removed the seasonal cycle and then detrend the long-term trends of soil moisture, vapor pressure deficit, precipitation, temperature and shortwave radiation to obtain the detrended data. The detrended data within ± 0.1 SD were considered in the normal state, those above 0.1 SD were considered positive anomalies, and those below -0.1 SD were considered negative anomalies. We calculated soil moisture during the recovery period as the cumulative sum of the difference between the detrended soil moisture and -0.1 SD, reflecting the overall deviation of soil moisture from the normal state [15]. According to the same method, we calculated vapor pressure deficit, temperature, precipitation and shortwave radiation during the recovery period of hot-dry and dry events. Then, we analysed the difference in DS between hot-dry and dry events, the difference in vegetation loss between hot-dry and dry events, and the differences in soil moisture, vapor pressure deficit, temperature, precipitation and shortwave radiation during recovery between hot-dry and dry events, written as ΔDS, Δloss, ΔSM, ΔVPD, ΔTEM, ΔPRE and ΔSrad, respectively.

A random forest regression model was used to explore the response of ΔRT to ΔDS, Δloss, ΔSM, ΔVPD, ΔTEM, ΔPRE and ΔSrad. We created a dataset with ΔRT as the dependent variable and ΔDS, Δloss, ΔSM, ΔVPD, ΔTEM, ΔPRE and ΔSrad as independent variables. Two-thirds of the data were used for model training, and one-third of the data were used for model validation. We finally built a random forest model consisting of 200 trees, with a leaf size of 5. A regression tree was applied to perform recursive binary splitting on the sample data [3], and variables that split more times were considered more important. Features importance is computed as the mean and standard deviation of accumulation of impurity decreases within each tree. Partial dependence plots were drawn to present the response curve of ΔRT to the independent variable.

## Stomatal and nonstomatal limitations caused by hot-dry and dry events derived from eddy covariance measurements

Productivity loss or greenness decline of vegetation result from stomatal and non-stomatal (maximum photosynthetic rate) limitations caused by droughts [16]. Stomatal limitation refers to the decline in photosynthesis caused by the partial closure of stomata during drought to save water [17], and non-stomatal restriction mainly refers to the decline in photosynthesis caused by non-stomatal factors such as the degradation of chloroplasts [18] and the declines in RuBP content and Rubisco activity [19]. We characterized the stomatal and non-stomatal limitation under hot-dry and dry events by using canopy conductance (*G_c_*) and the maximum photosynthetic assimilation rate (*A_max_*) derived from eddy covariance measurements from the FLUXNET2015 Tier 1 dataset [16,20]. To verify the consistency of FLUXNET2015 data with remote observations that hot-dry events led to a longer recovery time than dry events and that ΔRT was higher in drylands than in humid regions (Fig. S12), we used monthly temperature, precipitation and GPP (vegetation growth proxy) from the FLUXNET2015 dataset to identify hot-dry and dry events according to the following steps. We used observational records from 120 sites that lasted at least 5 years for the identification of hot-dry and dry events and then removed the seasonal cycle of temperature, precipitation and GPP by subtracting the multiyear monthly sample mean [8], resulting in deseasonalised data. We defined temperature, precipitation and GPP anomalies based on the SD of deseasonalised data, where deseasonalised GPP below -0.1 SD was considered a negative anomaly. To reflect the serious deviation of temperature and precipitation anomalies from their normal states, positive temperature anomalies were defined as deseasonalised temperature above 0.5 SD, and negative precipitation anomalies were defined as deseasonalised precipitation below -0.5 SD.

During the negative GPP anomaly period, the cooccurrence of negative precipitation anomalies and positive temperature anomalies was defined as hot-dry events, and during the negative GPP anomaly period, only negative precipitation anomalies without positive temperature anomalies were defined as dry events. Recovery time was defined as the time it took to recover from the minimum deseasonalised GPP to the normal state. We also assessed the recovery time from hot-dry and dry events identified by -0.1 SD as a negative precipitation anomaly and 0.1 SD as a positive temperature anomaly to enhance the robustness of the recovery time assessment at different thresholds (Fig. S13). The FLUXNET2015 dataset revealed the same findings as remote sensing observations that hot-dry events led to a longer recovery time than dry events, and the ΔRT between hot-dry and dry events was higher in drylands than in humid regions. On this basis, we further calculated the *G_c_* and *A_max_* [21,22] and presented the difference in *G_c_* anomalies and *A_max_* anomalies between hot-dry and dry events. The standardized anomalies of *G_c_* and *A_max_* were calculated according to Equations (4) and (5):

$G_{c} anomaly=\frac{G_{c}-\bar{G_{c}}}{\sigma(G_{c})}$ (4)

$A_{max} anomaly=\frac{A_{max}-\bar{A_{max}}}{\sigma\left( A_{max} \right)}$ (5)

where $\bar{G_{c}}$ and $\bar{A_{max}}$ are the mean values of *G_c_* and *A_max_* and $\sigma\left( G_{c} \right)$ and $\sigma\left( A_{max} \right)$ are the standard deviations of *G_c_* and *A_max_*.

Threshold values of the 10th, 20th, … and 90th percentiles of normalized vapor pressure deficit and soil moisture were used to bin *G_c_* and *A_max_* [23]. Specifically, we calculated the average *G_c_* and *A_max_* anomalies in different vapor pressure deficit and soil moisture percentiles and then plotted the response curve of average *G_c_* and *A_max_* to increased vapor pressure deficit percentiles and decreased soil moisture percentiles.

*G_c_* was calculated using half-hour data on rain-free days based on the inversion of the Penman‒Monteith equation [22], according to Equation 6.

$G_{c}=\frac{\frac{\lambda E\times\gamma}{r_{a}}}{\Delta\times\left( R_{n}-G \right)+\frac{\rho\times c_{p}\times\left( e_{s}-e_{a} \right)}{r_{a}}-\lambda E\times(\Delta+\gamma)}$ (6)

where *λE* refers to the fluxes of latent heat, *γ* is the psychrometric constant, *r_a_* is the aerodynamic resistance, *Δ* is the slope of the saturation vapour pressure curve, *R_n_* is the net radiation, *G* is the soil heat flux, *ρ* is the mean air density at constant pressure, *c_p_* is the air specific heat at constant pressure, *e_s_* is the saturation vapour pressure and *e_a_* is the actual vapour pressure.

*γ* is calculated according to Equation 7, where *c_p_* is the air specific heat at constant pressure, 1013 (J kg^-1^ ℃^-1^); *P* is the atmosphere pressure (kPa); ε is the ratio molecular weight of water vapor/dry air, 0.622; and *λ* is the latent heat of vaporization of water (kJ kg^-1^).

$\gamma=\frac{c_{p}\times P}{\varepsilon\times\lambda}$ (7)

*λ* is calculated according to Equation 8, where *T* is the mean air temperature (℃).

$\lambda=2500-2.37\times T$ (8)

*r_a_* is calculated according to Equation 9 [24], where *k* is the von Kármán constant, 0.41; *z_m_* is the measurement height; *z_0_* is the momentum roughness length, *z_0_* =0.1 *h*; *z_d_* is the zero plane displacement, *z_d_* = 0.67 *h*; and *h* is the canopy height calculated by Equation 10 [25].

$r_{a}=\frac{{\ln\left( \frac{z_{m}-z_{d}}{z_{0}} \right)}^{2}}{w_{s}{\times k}^{2}}$ (9)

$h=\frac{z_{m}}{0.6+0.1\times exp(\frac{{k\times w}_{s}}{u})}$ (10)

*Δ* is calculated according to Equation 11

$\Delta=\frac{4098\times(0.6108\times exp(\frac{17.27\times T}{T+237.3}))}{{(T+237.3)}^{2}}$ (11)

*A_max_* is derived from non-gap-filled net carbon flux (*Fc*) measurements [21]. *Fc* represents the balance of CO_2_ taken up for photosynthesis and released via respiration and is calculated according to Equation 12 [21], where α is the canopy-scale quantum yield; β is the maximum rate of CO_2_ uptake of the canopy under saturating light levels, equivalent to *A_max_*; *R_g_* is the global radiation; and *R_e_* is ecosystem respiration.

$F_{c}=\frac{\alpha\times\beta\times R_{g}}{\alpha\times R_{g}+\beta}+R_{e}$ (12)

The impact of vapor pressure deficit on β is shown in Equation 13, in which β decreases exponentially with increasing vapor pressure deficit when vapor pressure deficit exceeds a threshold (VPD_0_). *β_0_* and *k* are fitted parameters, and VPD_0_ is 1 kPa. Daily *A_max_* was calculated by Equations 12 and 13 using the REddyProc R package (<https://github.com/bgctw/REddyProc>) [26].

$\beta=\left\{ \begin{aligned} \beta_{0}\exp\left( -k\left( VPD-{VPD}_{0} \right) \right), VPD>{VPD}_{0} \\ \beta_{0}, VPD\leq{VPD}_{0} \end{aligned} \right.$ (13)

# Supplementary figures


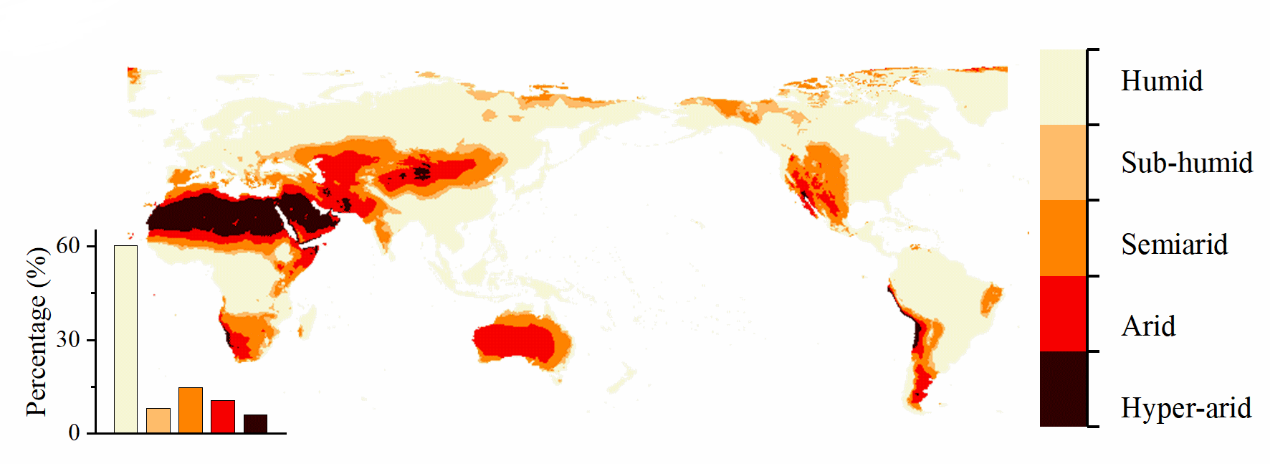


Fig. S1 Global distribution of dryland (0<AI≤0.65) and humid region (AI>0.65) identified by aridity index (the ratio of precipitation to potential evapotranspiration). Dryland includes hyper-arid (AI≤0.05), arid (0.05<AI≤0.2), semiarid (0.2<AI≤0.5) and sub-humid (0.5<AI≤0.65) region.


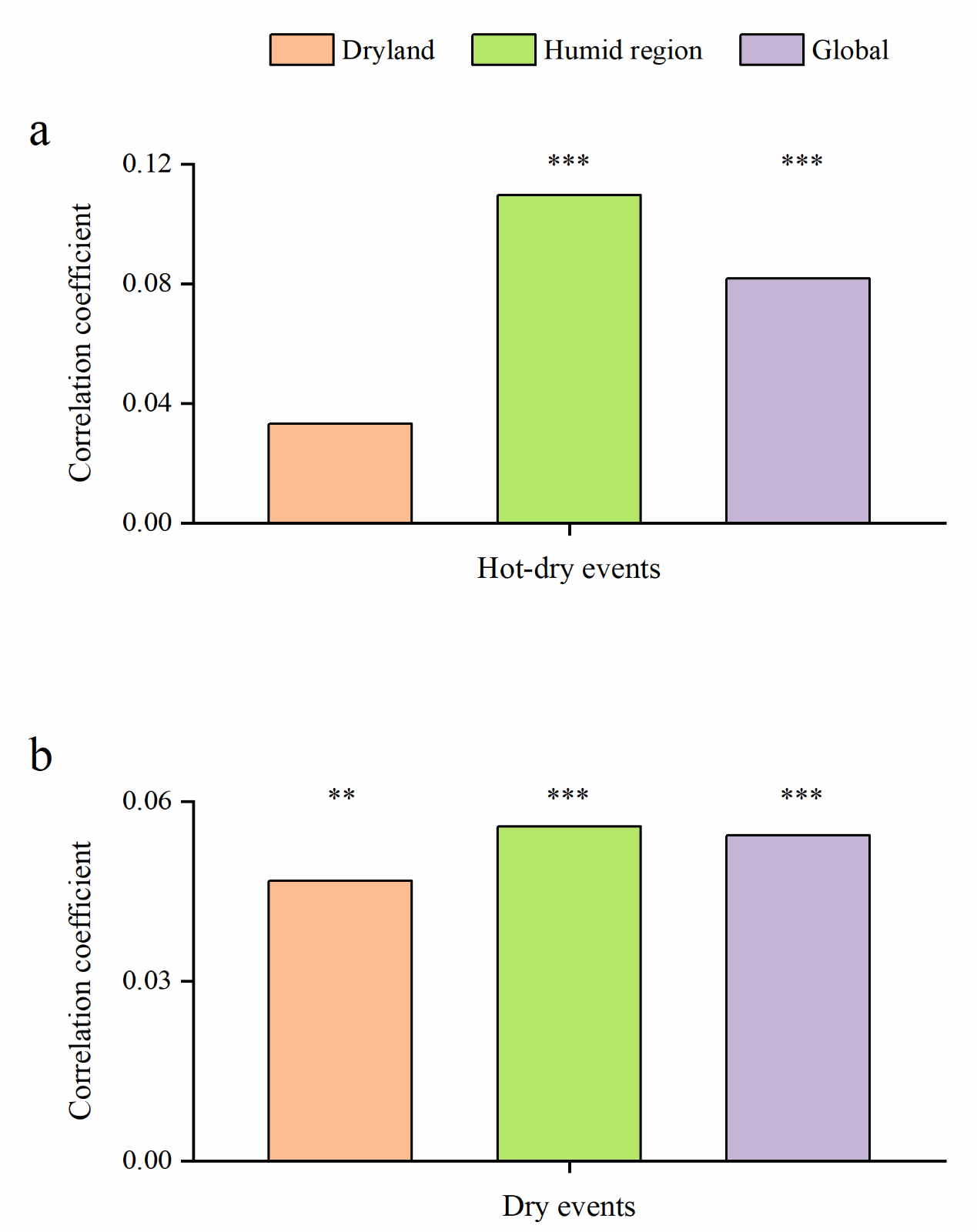
Fig. S2 Correlation coefficients between recovery time and canopy height. *a*, hot-dry events. *b*, dry events. Asterisks indicate statistically significant differences (*** P < 0.001; ** P < 0.05).


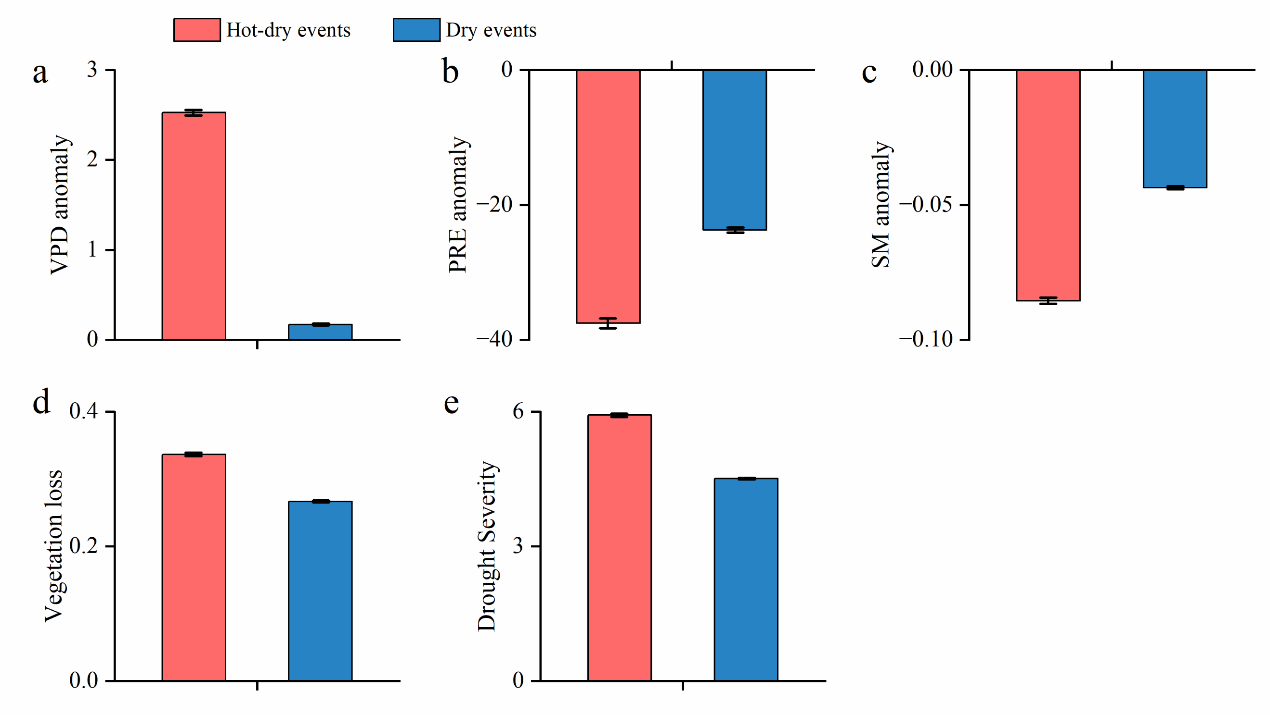


Fig. S3 *a*, *b* and *c*, vapour pressure deficit, precipitation and soil moisture anomaly during the recovery period, respectively. *d*, vegetation loss; *e*, drought severity.


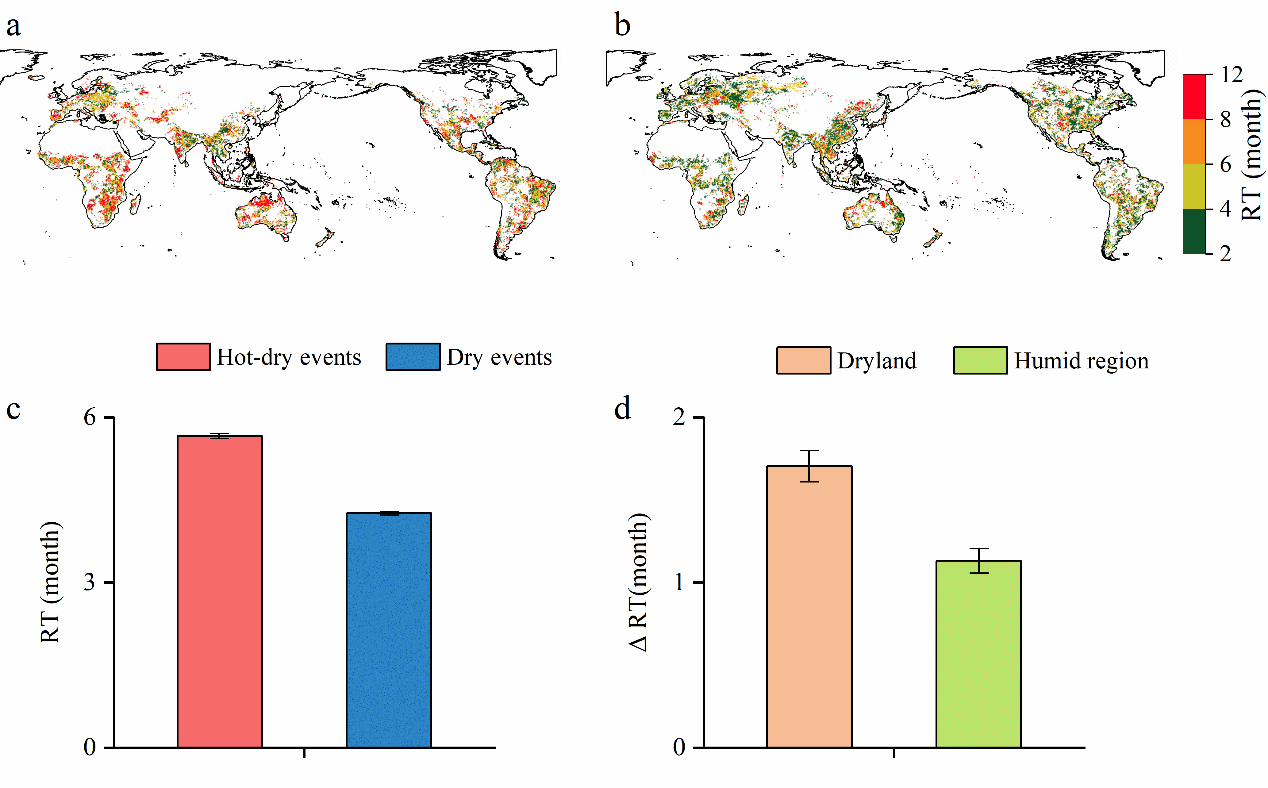


Fig. S4 Recovery time of ecosystems to hot-dry and dry events based on VOD. *a*, spatial pattern of recovery time to hot-dry events. *b*, spatial pattern of recovery time to dry events. *c*, comparison between recovery time of ecosystems to hot-dry and dry events. *d*, ΔRT (the difference in recovery time of ecosystems to hot-dry and dry events) in dryland and humid region.


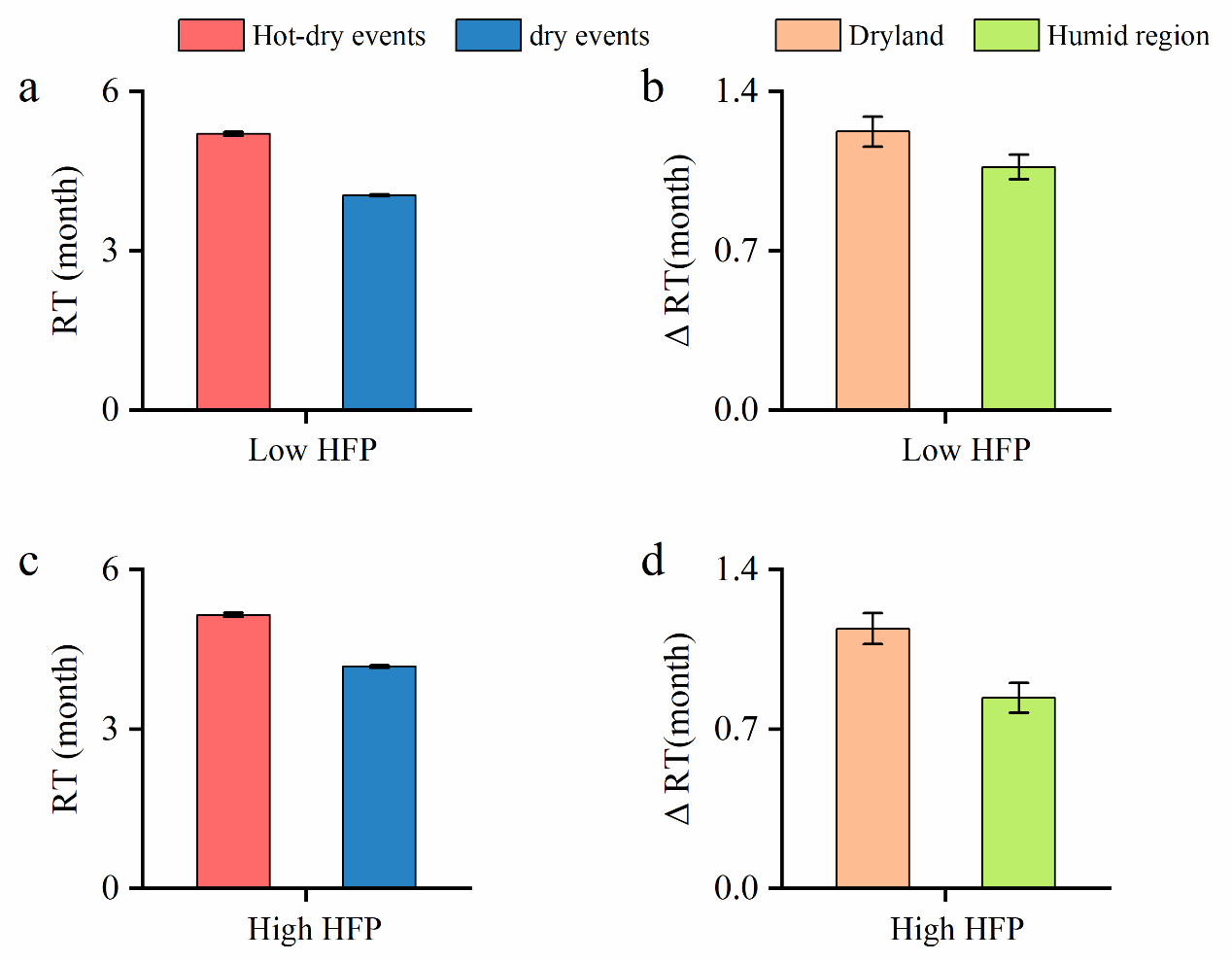
Fig. S5 Recovery time under different human footprints. *a*, *c*, the recovery time of ecosystem to hot-dry and dry events under low and high human footprint, respectively. *b*, *d*, ΔRT (the difference in recovery time between hot-dry and dry events) in dryland and humid region under low and high human footprint, respectively.


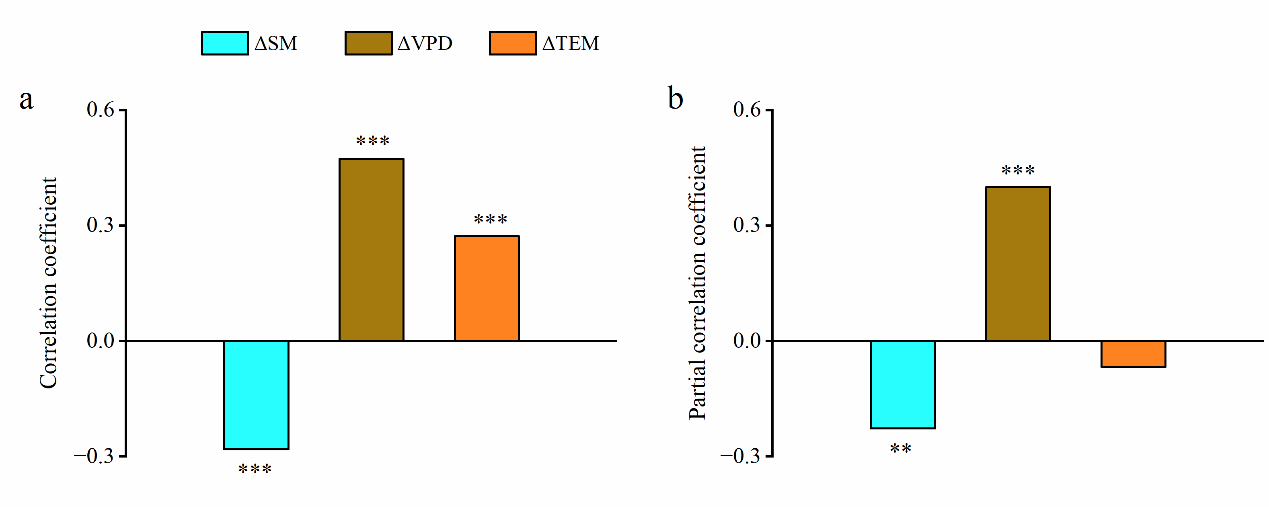


Fig. S6 *a*, correlation coefficients between ΔRT and ΔVPD, ΔTEM, and ΔSM, respectively. *b*, partial correlation coefficients between ΔRT and ΔVPD, ΔTEM, and ΔSM, respectively. Asterisks indicate statistically significant differences (*** P < 0.001; ** P < 0.05).


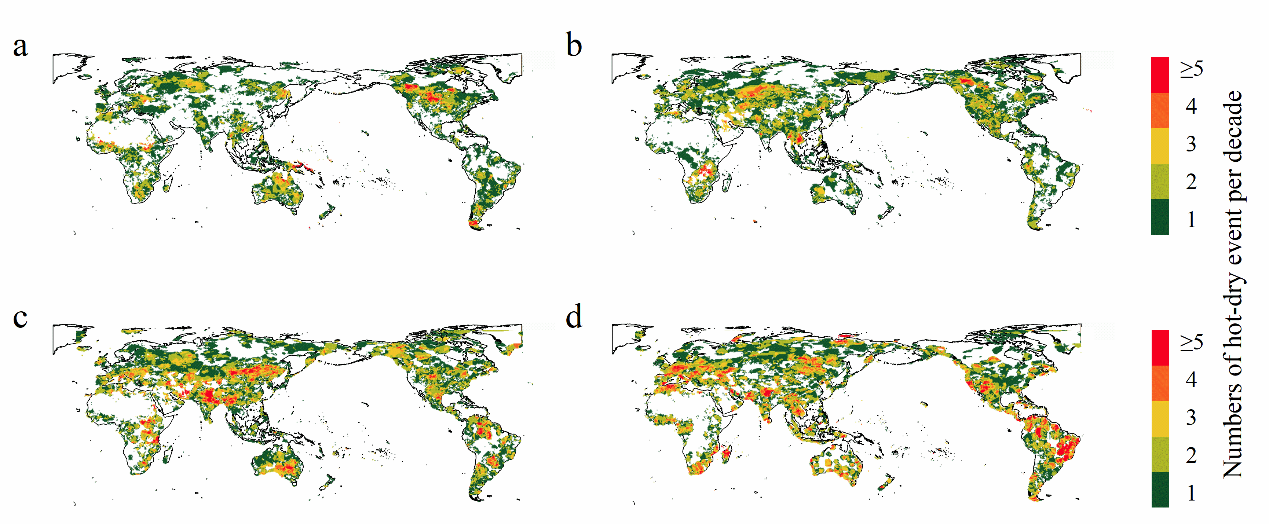


Fig. S7 Numbers of hot-dry events per decade. *a*, *b*, *c* and *d* are the 1980s, 1990s, 2000s, and 2010s, respectively.


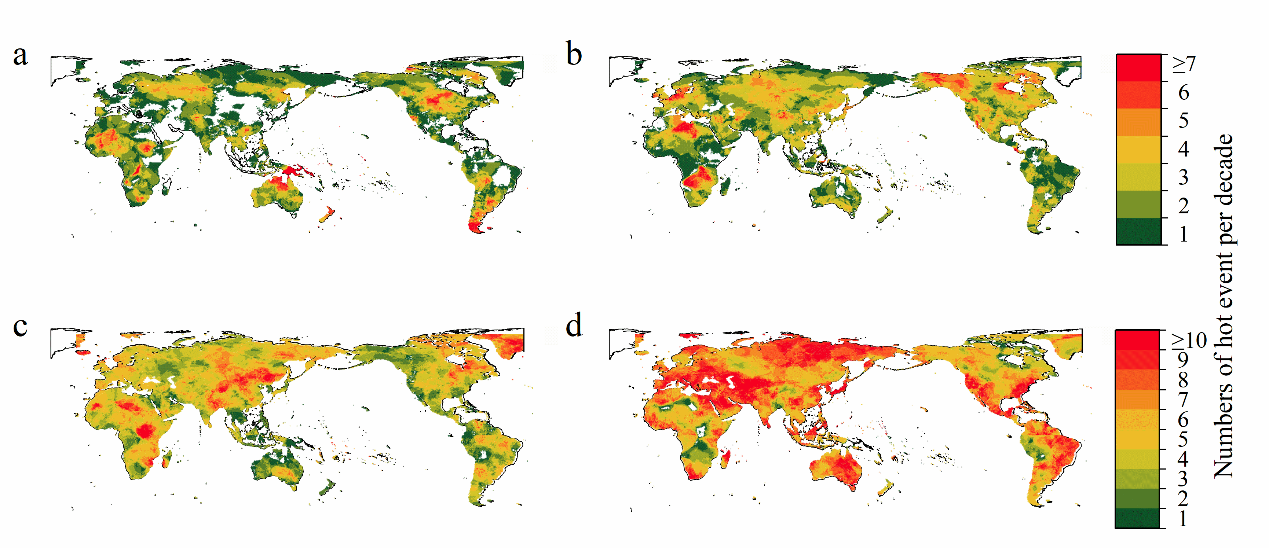


Fig. S8 Numbers of hot events per decade. *a*, *b*, *c* and *d* are the 1980s, 1990s, 2000s, and 2010s, respectively.



Fig. S9 *a*, Raw values of the NDVI for 30 consecutive months and *b*, Deseasonalised NDVI data during 30 consecutive months. The red asterisks indicate the state of vegetation in February for three consecutive years.


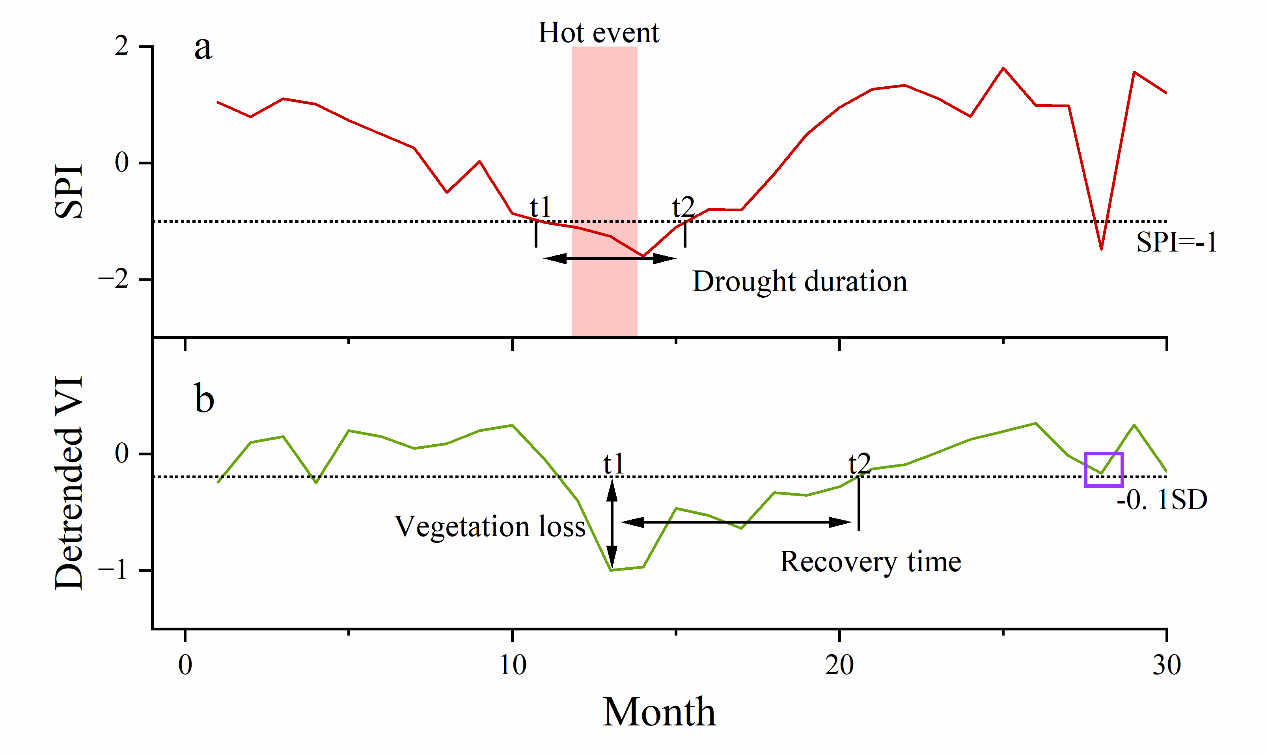


Fig. S10 Identification of hot-dry events, drought duration, and recovery time. *a*, t1 and t2 represent the start and end of the hot-dry event, and the length of time between them is defined as the drought duration. *b*, t1 and t2 represent the start and end of recovery, respectively, and the length of time between them is defined as the recovery time. Purple highlighting indicates that detrended vegetation index (VI) is below 0 but greater than -0.1SD.


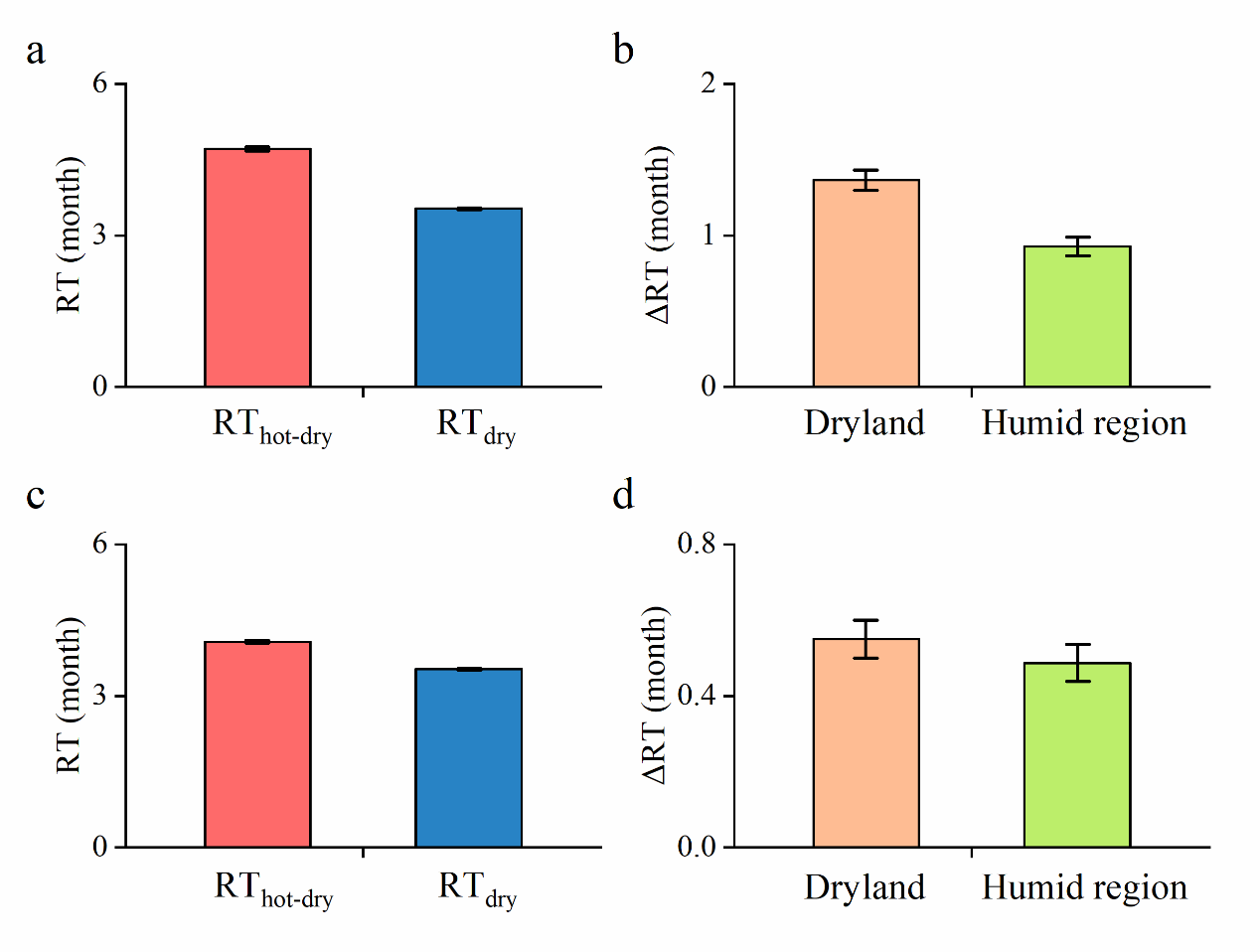


Fig. S11 Recovery time based on a negative vegetation anomaly threshold of -0.5 SD. *a*, The average recovery times based on NDVI from hot-dry and dry events are 4.71 and 3.53 months. *b*, the ΔRTs based on NDVI are 1.36 and 0.93 months in drylands and humid regions, respectively. *c*, the average recovery times based on LAI from hot-dry and dry events are 4.07 and 3.53 months. *d*, the ΔRT based on LAI is 0.55 and 0.49 months in drylands and humid regions, respectively.





Fig. S12 Distribution of FLUXNET sites used in this study. *a*, the spatial distribution of FLUXNET sites, *b*, the proportion of sites in dryland and humid region, and *c*, the proportion of sites with different vegetation types.


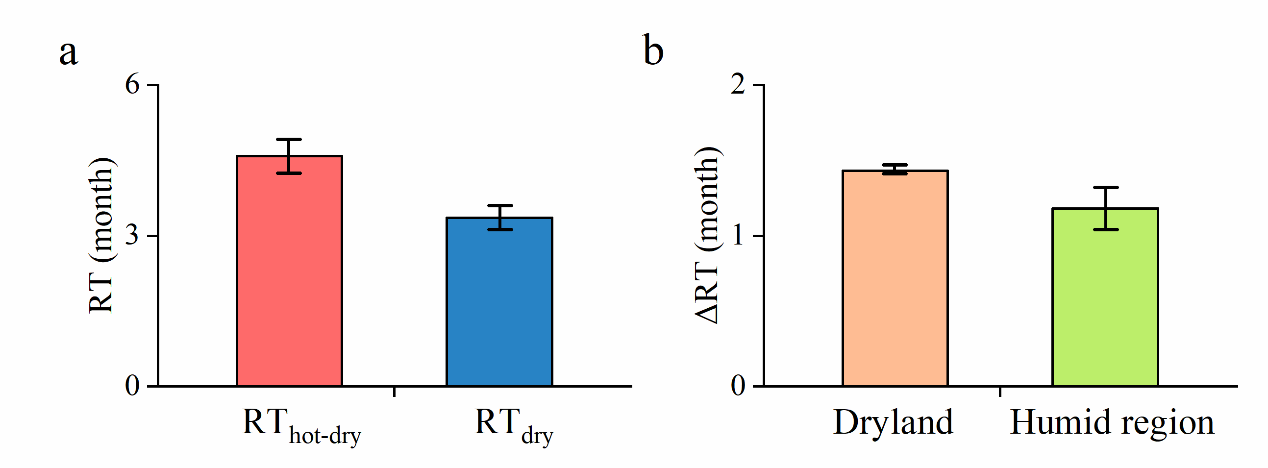
Fig. S13 Recovery time from hot-dry and dry events identified by -0.1 SD as a negative precipitation anomaly and 0.1 SD as a positive temperature anomaly based on eddy covariance measurements from the FLUXNET2015 dataset. *a*, the average recovery times from hot-dry and dry events is 4.58 and 3.36 months. *b*, the average ΔRT is 1.43 and 1.18 months in drylands and humid regions, respectively.

# References

1. Guan YL, Liu JL and Chen AF *et al*. Spatial Aggregation of Global Dry and Wet Patterns Based on the Standard Precipitation Index. *Earth Future* 2022; **10**: e2022EF002720.

2. Vicente-Serrano SM, Beguería S and López-Moreno JI. A Multiscalar Drought Index Sensitive to Global Warming: The Standardized Precipitation Evapotranspiration Index. *J. Clim.* 2010; **23**: 1696–1718.

3. Zhang Y, Keenan TF and Zhou S. Exacerbated drought impacts on global ecosystems due to structural overshoot. *Nat. Ecol. Evol.* 2021; **5**:1490–8.

4. Zhang Y and Li ZL. Uncertainty Analysis of Standardized Precipitation Index Due to the Effects of Probability Distributions and Parameter Errors. *Front. Earth Sci.* 2020; **8**: 76.

5. Zhu ZC, Bi J and Pan YZ *et al*. Global Data Sets of Vegetation Leaf Area Index (LAI)3g and Fraction of Photosynthetically Active Radiation (FPAR)3g Derived from Global Inventory Modeling and Mapping Studies (GIMMS) Normalized Difference Vegetation Index (NDVI3g) for the Period 1981 to 2011, *Remote Sens.* 2013; **5**: 927:48.

6. Jiao WZ, Wang LX and Smith WK *et al*. Observed increasing water constraint on vegetation growth over the last three decades. *Nat. Commun.* 2021; **12**: 3777.

7. Zhang SL, Yang YT and Wu XC *et al*. Postdrought Recovery Time Across Global Terrestrial Ecosystems. *J. Geophys. Res.-Biogeosci.* 2021; **126**: e2020JG005699.

8. Forzieri G, Dakos V and McDowell NG *et al*. Emerging signals of declining forest resilience under climate change. *Nature* 2020; **608**: 534–9.

9. Jiao T, Williams CA and De Kauwe MG *et al*. Patterns of post‐drought recovery are strongly influenced by drought duration, frequency, post‐drought wetness, and bioclimatic setting. *Glob. Change Biol.* 2021; **27**: 4630–43.

10. Yao N, Li Y and Lei TJ *et al*. Drought evolution, severity and trends in mainland China over 1961–2013. *Sci. Total Environ*. 2018; **616–617**: 73–89.

11. Bevacqua E, Zappa G and Lehner F *et al*. Precipitation trends determine future occurrences of compound hot–dry events. *Nat. Clim. Chang.* 2022; **12**: 350–5.

12. Huang JP, Yu HP and Guan XD *et al*. Accelerated dryland expansion under climate change. *Nat. Clim. Chang.* 2016; **6**: 166–71.

13. Schwalm CR, Anderegg WRL and Michalak AM *et al*. Global patterns of drought recovery. *Nature* 2017; **548**: 202–5.

14. He B, Liu JJ and Guo LL *et al*. Recovery of Ecosystem Carbon and Energy Fluxes From the 2003 Drought in Europe and the 2012 Drought in the United States. *Geophys. Res. Lett.* 2018; **45**: 4879–88.

15. Chiang F, Mazdiyasni O and AghaKouchak A. Evidence of anthropogenic impacts on global drought frequency, duration, and intensity. *Nat. Commun.* 2021; **12**: 2754.

16. Fu Z, Ciais P and Prentice IC *et al*. Atmospheric dryness reduces photosynthesis along a large range of soil water deficits. *Nat. Commun.* 2022; **13**: 989.

17. Henry C, John GP and Pan RH *et al*. A stomatal safety-efficiency trade-off constrains responses to leaf dehydration. *Nat. Commun.* 2019; **10**: 3398.

18. Wang YX, Li XY and Liu NN *et al*. The iTRAQ-based chloroplast proteomic analysis of Triticum aestivum L. leaves subjected to drought stress and 5-aminolevulinic acid alleviation reveals several proteins involved in the protection of photosynthesis. *BMC Plant Biol.* 2020; **20**: 96.

19. Bota J, Medrano H and Flexas J. Is photosynthesis limited by decreased Rubisco activity and RuBP content under progressive water stress? *New Phytol.* 2004; **162**: 671–81.

20. Wilson KB, Baldocchi DD and Hanson PJ. Quantifying stomatal and non-stomatal limitations to carbon assimilation resulting from leaf aging and drought in mature deciduous tree species. *Tree Physiol.* 2000; **20**: 787–97.

21. Luo XZ and Keenan TF. Global evidence for the acclimation of ecosystem photosynthesis to light. *Nat. Ecol. Evol.* 2020; **4**: 1351–7.

22. dos Reis MG and Ribeiro A. Conversion factors and general equations applied in agricultural and forest meteorology. *AgroM.* 2019; **27**: 227–58.

23. Liu LB, Gudmundsson L and Hauser M *et al*. Soil moisture dominates dryness stress on ecosystem production globally. *Nat. Commun.* 2020; **11**: 4892.

24. Novick KA, Ficklin DL and Stoy PC *et al*. The increasing importance of atmospheric demand for ecosystem water and carbon fluxes. *Nat. Clim. Chang.* 2016; **6**: 1023–7.

25. Pennypacker S and Baldocchi D. Seeing the Fields and Forests: Application of Surface-Layer Theory and Flux-Tower Data to Calculating Vegetation Canopy Height. *Bound.-Layer Meteor.* 2016; **158**: 165–82.

26. Lasslop G, Reichstein M and Papale D *et al*. Separation of net ecosystem exchange into assimilation and respiration using a light response curve approach: critical issues and global evaluation: SEPARATION OF NEE INTO GPP AND RECO. *Glob. Change Biol.* 2010; **16**: 187–208.
